# Aberrations in peripheral B lymphocytes and B lymphocyte subsets levels in Parkinson disease: a systematic review

**DOI:** 10.3389/fimmu.2025.1526095

**Published:** 2025-03-31

**Authors:** Hongxia Ma, Ziyuan Wang, Miao Yu, Yibo Zhai, Junqiang Yan

**Affiliations:** ^1^ Department of Neurology, The First Affiliated Hospital, College of Clinical Medicine of Henan University of Science and Technology, Luoyang, China; ^2^ Key laboratory of Neuromolecular Biology, The First Affiliated Hospital, College of Clinical Medicine of Henan University of Science and Technology, Luoyang, China

**Keywords:** B lymphocyte, B lymphocyte subsets, Parkinson’s disease, Pathogenesis, Immunity, Inflammation

## Abstract

**Objective:**

The association of B lymphocytes and B lymphocyte subsets and Parkinson’s disease (PD) is increasingly acknowledged. However, there is inconsistence in the alterations of B lymphocytes or B lymphocyte subsets in peripheral blood of PD patients. To comprehensively understand its changes in PD patients,it is necessary to conduct a systematic review on this subject.

**Methods:**

PubMed, Cochrane Library, and MEDLINE databases were searched until 3^rd^ February 2024.

**Results:**

We included 20 studies (n=2658) to conduct this systematic review. We conducted a qualitative analysis to assess the alterations of B lymphocytes and B lymphocyte subsets in the peripheral blood of individuals with PD. And studies reviewed demonstrated a significant decrease in the number of B cells, as well as immune dysregulation in the B lymphocyte subsets of these patients’ peripheral blood.

**Conclusion:**

Studies reviewed demonstrated that PD is linked to abnormalities in B lymphocytes and/or B lymphocytes subsets in peripheral blood. This study provides a novel perspective into the pathogenesis of PD, and future investigations into the B lymphocytes and/or B lymphocyte subsets as biomarkers and therapeutic targets for PD is warranted.

## 1 Introduction

Parkinson’s Disease(PD), the second most common progressive neurodegenerative disorder, causes motor and non-motor symptoms. PD is not common among individuals younger than 50 years and increases in prevalence with age (1). One data estimated by The Global Burden of Disease Study has shown that the prevalence of PD has reached 11.8 million in 2021, with a percentage change of 273.9%, which may bring a tremendous burden to society (2).

The characteristic of PD is the death of dopaminergic neurons in the substantia nigra. The pathological feature of PD is the Lewy body, which is a neuronal inclusion consistinglargely of α-synuclein protein aggregations (3). Although decades of research and the development of a large group of animal models, our understanding of the mechanisms responsible for the progressive loss of dopamine neurons in PD is still unclear (4). Research has shown that IgG antibodies deposit on dopaminergic neurons in Parkinson’s disease patients, and the Lewy bodies were also coated by IgG (5). Although B lymphocytes were not detected in the brains of PD patients (6), these results were later supported by animal model studies of PD, revealing long-term infiltration of B cells in the midbrain of rodents (7–9) and non-human primates (10, 11). Therefore, the role of B lymphatic system in the occurrence and development of PD is receiving increasing attention (12–16).

B lymphocytes, as a part of the adaptive immune system, play multiple roles and have complex interactions with other branches of the innate and adaptive immune systems (17). A simple definition of B lymphocytes is a population of cells that express clonally diverse cell surface immunoglobulin (Ig) receptors which can recognize specific antigenic epitopes (18). B lymphocytes are crucial for normal immune system development and its maintenance (18). In addition to producing antibodies, B lymphocytes also can release immune regulatory cytokines, antigen-presenting cell functions, and regulate T cells and the innate response (17, 18). Studies have shown that after the death of dopaminergic neurons, their antigens are presented to the immune system, with activation of B lymphocytes. Then, B lymphocytes or specific autoantibodies might enter the central nervous system through the dysfunctional blood brain barrier, produce cytokines that activate microglia, and release autoantibodies. This may lead to further inflammation and subsequent cell death (4).

Inflammation and aberrant immune responses also play a crucial role in the development of PD (19, 20). Regulatory B cells (Bregs), a subpopulation of lymphocytes, have been associated with the inhibition of excessive inflammation and play a crucial role in maintaining immune homeostasis. Dysfunction of Bregs has been observed in PD. Immune dysfunction has become a research hotspot in the pathogenesis and progression of PD. In recent years, domestic and foreign researchers have further explored immunophenotype of peripheral blood B lymphocytes in Parkinson’s disease (12–16). These results indicate that the alterations of B lymphocytes or B lymphocyte subsets in peripheral blood are associated with the progression of PD (14, 21, 22). These peripheral abnormalities may drive the occurrence and progression of PD by enhancing immune cell infiltration and enhancing neuroinflammation in the central nervous system (23). Although many studies have shown an association between B lymphocytes (or B lymphocyte subsets) and PD, these results are inconsistent in the alterations of B lymphocytes or B lymphocyte subsets in peripheral of PD patients. To comprehensively understand the changes in B lymphocytes or B lymphocyte subsets in PD patients, and to provide crucial evidence for studying the mechanisms underlying this illness, it is necessary to conduct a systematic review on this subject. Moreover, the precise nature of the changes in B lymphocytes or B lymphocyte subsets in peripheral of PD patients remain unclear. Consequently, a systematic review of studies of various B lymphocyte subsets in PD is also required for a greater understanding of the nature of immune dysfunction in this illness.

## 2 Methods

### 2.1 Search strategy

Studies published before 3^rd^ February 2024 were searched in PubMed, Cochrane Library, and MEDLINE databases. Standard keyword searches were conducted using “Parkinson’s disease”, “B lymphocytes”, “CD19^+^ B cells”, “CD20^+^ B cells”, “transitional B cells”, “regulatory B cells (Bregs)”, “naïve B cells”, “memory B cells”or “plasma cells”. Two authors (H-X, M and Z-Y, W) screened titles and abstracts independently and relevant studies were retrieved. Electronic search has been supplemented by hand-searching meta-analysis and review articles. Any differences were resolved through further discussion. The detailed search strategy for each database is presented in the online **Supplementary Materials**. This review and protocol was prospectively registered on PROSPERO (registration No. CRD42024508329) and conducted following the PRISMA’s Preferred Reporting Items guidelines for Systematic Reviews and Meta-analyses Protocols (24). The PRISMA checklist is presented in **Supplementary Table 1**.

### 2.2 Inclusion and exclusion criteria

The research question of our systematic review clearly defined in terms of populations, interventions, comparators, outcomes, and study designs (PICOS): (1) Study type (S): case-control or cohort in design; (2) Participants (P): patients were clinically diagnosed with PD (patients who were on immunomodulatory treatments or had recent vaccinatinos or infections have been excluded); (3)Interventions (I): used flow cytometry, single cell RNA-seq, or related techniques to identify and describe peripheral blood B lymphocytes phenotype; (4) Comparison (C): healthy individuals; (5) Outcome (O): at least one of the following endpoints (the percentage of B lymphocyte subsets or absolute counts of B lymphocyte in peripheral blood) was reported, including CD19^+^ B cells, CD20^+^ B cells, transitional B cells, regulatory B cells, naïve B cells, memory B cells, and plasma cells.

Exclusion criteria: (1) Not written in English; (2) Duplicate publication; (3) Case report; (4) Studies that did not report B lymphocyte count or phenotype were excluded.

### 2.3 Data extraction

The following data were collected from each included study: (1) Baseline characteristics: including first author’s name, year of publication, country, sample size, sex ratio, H&Y score, sampling source, case definition, control definition, immune cells assessed, and Newcastle-Ottawa Scale (NOS) Quality scores; (2) Mean and Standard deviation (SD) of the counts or percentage of B lymphocyte and B lymphocyte subsets in peripheral blood in PD patients and controls; (3) Individual immune cell types and cell classifications used in the current investigation (**Supplementary Table 2**). Two researchers (M, Y and Y-B, Z) were responsible for extracting the necessary data for this meta-analysis and systematic review. In cases where the required data were not reported, we would contact the author to obtain this data. Any disagreements or differences were resolved through further discussion with a third independent researcher (H-X, M), if necessary.

### 2.4 Assessment of methodological quality

The methodological quality of each included study was assessed independently by two authors (M, Y and Y-B, Z), and any disagreements were resolved through further discussion led by another author (H-X, M). We employed an adapted version of the NOS for case-control studies (25) to assess the methodological quality. The non-response rate, one NOS scoring item, was considered irrelevant to the current investigation, so the maximum possible NOS score here is was 8. A score of 5-8 indicates high-quality research.

## 3 Results

### 3.1 Selected studies and study characteristics

**Figure 1:**
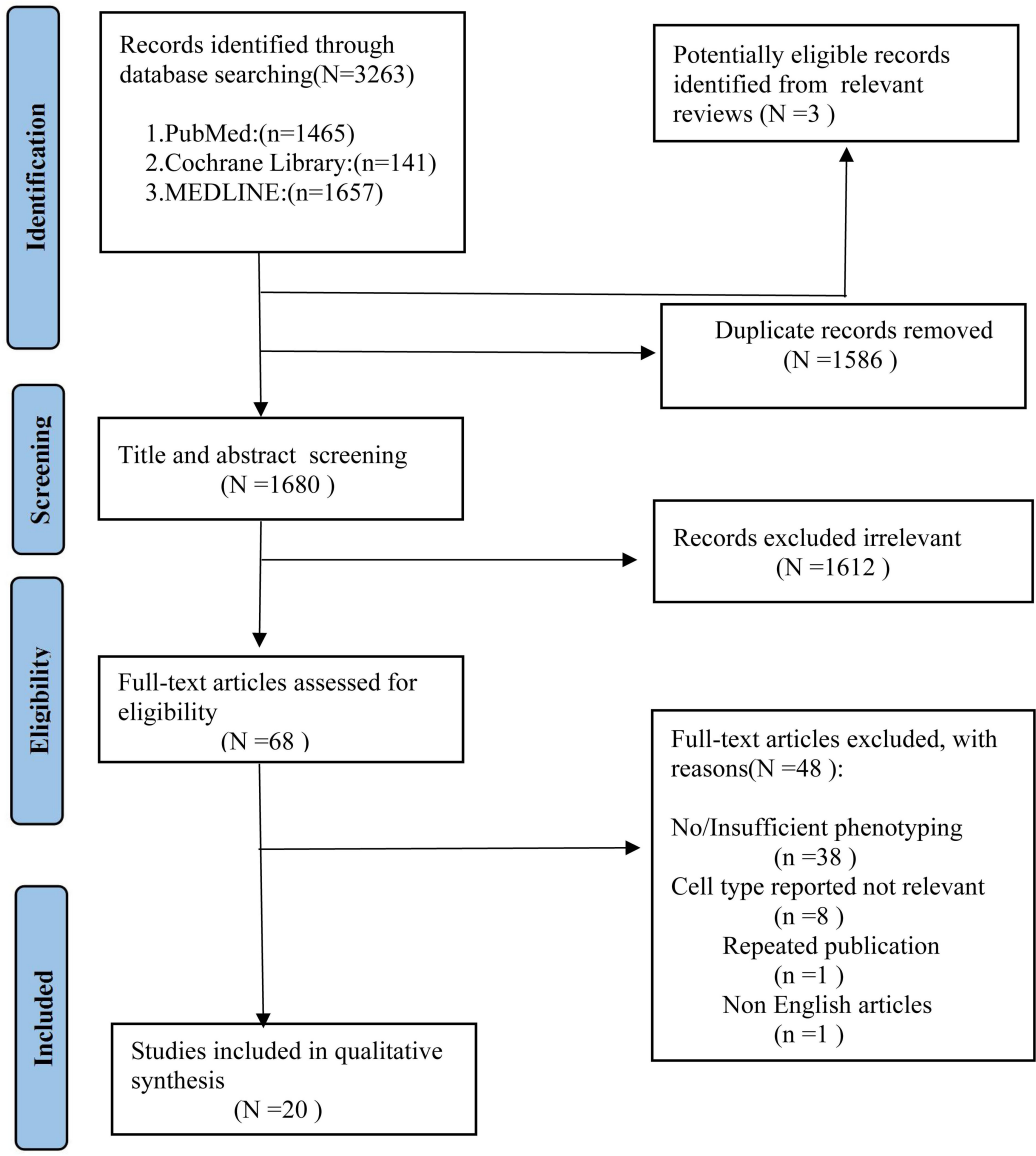
PRISMA flow diagram for study selection.

As shown in **Figure 1**, we identified 3263 records from PubMed, Cochrane Library, MEDLINE databases. After excluding the duplicates studies (1586 records) in EndNote, the irrelevant studies (1612 records) were removed through reading titles and abstracts and the remaining 68 articles needed us to read the full text to identify available data. 48 articles were excluded. Ultimately, a total of 20 studies encompassing 2658 unique study participants, including 1407 PD patients and 1251 healthy controls, were included in the systematic review. The basic characteristics of included studies were presented in **Table 1**.

**Table 1:** Basic characteristics of included studies in systematic review.

No.	Author	Year	Country	Sample Size	Gender(M/F)	Age in years (mean ± SD)	H&Y score (mean± SD)	Sampling source	Case definition	Control definition	Immune cells assessed	NOS scores
1	Claire H Stevens et al.	2012	Australia	Parkinson’s disease (n=88) Controls (n=77)	Parkinson’s disease (56/32) Controls (n=39/38)	Parkinson’s disease (69 ± 9) Controls (67 ± 10)	2 ± 0.7	Cases: Not specified Controls: Not specified	All PD patients had a tremor-dominant phenotype and were levodopa responsive. No patient had a haematological, immune or inflammatory disorder, or was taking immunosuppressive medications. Any patient with a neurologic or psychiatric condition other than PD, strong family history of PD or relative with young onset PD was excluded.	No control subject had a haematological, immune or inflammatory disorder, or was taking immunosuppressive medications.	CD19+ B cells	6
2	Jordi Bas et al.	2001	Spain	Untreated Parkinson’s disease (n=30) Treated Parkinson’s disease (n=34) Controls (n=38)	Parkinson’s disease Not reported Controls Not reported	Untreated Parkinson’s disease (66 ± 11) Treated Parkinson’s disease (65 ± 9) Controls (63 ± 4)	Not reported	Cases: Hospital inpatients Controls: Community	fulfill diagnostic criteria of idiopathic Parkinson’s disease and the absence of central nervous system lesions by a CT scan. The exclusion criteria were the presence of diseases that could affect significantly the immunological parameters assessed, such as inflammatory processes, autoimmune diseases and neoplasia.	healthy blood donors	CD19+ B cells	6
3	Fumitoshi Niwa et al.	2012	Japan	Parkinson’s disease (n=29) Controls (n=30)	Parkinson’s disease (17/12) Controls (n=16/14)	Parkinson’s disease (70.42 ± 7.23) Controls (68.93 ± 5.12)	2.63 ± 0.92	Cases: Hospital inpatients Controls: Community	Patients diagnosed with sporadic PD according to the United Kingdom Parkinson’s Disease Society Brain Bank criteria. Patients diagnosed with other forms of neurodegenerative Parkinsonism, cerebrovascular Parkinsonism or neuropsychogenic disorders such as depression or drug-induced Parkinsonism were excluded. All subjects had normal findings on brain magnetic resonance imaging, with no apparent cerebrovascular lesions or past history of neurological abnormalities. Subjects suffering from dysphagia or loss of appetite were excluded from the study to minimize the influence of immunological conditions.	Subjects had normal findings on brain magnetic resonance imaging, with no apparent cerebrovascular lesions or past history of neurological abnormalities.	CD20+ B cells	7
4	Kirsten M Scott et al.	2023	UK	Parkinson’s disease (n=41) Controls (n=41)	Parkinson’s disease (28/13) Controls (n=28/13)	Parkinson’s disease (68.4 ± 6.3) Controls (68.1 ± 5.6)	Not reported(≤ 2)	Cases: Hospital outpatients Controls: Community	Parkinson’s disease cases were fulfilment of the UK Parkinson’s disease Brain Bank Criteria. No neurodegenerative disorders, chronic inflammatory/autoimmune disorders, current infection, surgery in the last month, vaccinations in the previous 3 weeks or recent use of anti-inflammatory/immunomodulating medications.	No history of neurological disease, memory problems or depression. No neurodegenerative disorders, chronic inflammatory/autoimmune disorders, current infection, surgery in the last month, vaccinations in the previous 3 weeks or recent use of anti-inflammatory/immunomodulating medications.	CD19+ B cells, transitional B cells, naïve B cells, CSM B cells, DNM B cells,USM B cells,plasma cells, regulatory B cells(CD1d+)	8
5	Pingping Wang et al.	2022	China	Parkinson’s disease (n=8) Controls (n=6)	Parkinson’s disease (4/4) Controls (n=4/2)	Parkinson’s disease Not reportedControls Not reported	Not reported(HY=2)	Cases: Hospital Controls: Hospital	No obvious somatic disorders, such as cancer, autoimmune diseases, as well as mental and cognitive disorders.	No obvious somatic disorders, such as cancer, autoimmune diseases, as well as mental and cognitive disorders.	CD19+ B cells, naïve B cells, USM B cells, CSM B cells,plasmablast/plasma cells	6
6	Marina A. Gruden et al.	2011	Russia	Parkinson’s disease (n=32) Controls (n=26)	Parkinson’s disease (20/12) Controls (19/7)	Parkinson’s disease (60.8 ± 2.0) Controls (63.0 ± 3.0)	2.1 ± 0.6	Cases: Hospital inpatients Controls: Not specified	Patients underwent neurological examination and were diagnosed with PD according to disease severity by the UPDRS. No opportunistic infections.	No opportunistic infections	CD20+ B cells	6
7	Rui Li et al.	2022	USA	Discovery Cohort: Parkinson’s disease (n=12) Controls (n=17) Validation Cohort: Parkinson’s disease (n=18) Controls (n=18)	Discovery Cohort: Parkinson’s disease 11/1) Controls (11/6) Validation Cohort: Parkinson’s disease (13/5) Controls (13/5)	Discovery Cohort: Parkinson’s disease 73.7 ± 6.4) Controls (67.8 ± 6.2) Validation Cohort: Parkinson’s disease (64.3 ± 10.6) Controls (61.1 ± 11.6)	Not reported	Cases: Hospital inpatients Controls: Hospital inpatients	All individuals with PD met the diagnostic criteria of the United Kingdom Parkinson’s Disease Brain Bank as previously described for these clinical cohorts. None of the participants had any suggestion of acute or chronic infection or on any immune-modifying therapy.	All normal controls had no known neurologic disorder. None of the participants had any suggestion of acute or chronic infection or on any immune-modifying therapy.	CD19+ B cells, transitional B cells, USM B cells, CSM B cells, Naïve B cells, DNM B cells, plasma cells	7
8	Luan Cen et al.	2017	China	Parkinson’s disease (n=268 Controls (n=268)	Parkinson’s disease (156/112) Controls (168/100)	Parkinson’s disease (60.59 ± 11.112) Controls (59.41 ± 11.113)	1.903 ± 0.865	Cases: Hospital outpatients Controls: Hospital outpatients	PD patients were diagnosed using the UK Brain Bank clinical criteria. No autoimmune or inflammatory disorder and those receiving chronic immunosuppressive therapy.	Age- and gender-matched controls were identified during routine health examinations. No autoimmune or inflammatory disorder and those receiving chronic immunosuppressive therapy.	CD19+ B cells	7
9	Xudong Zhao et al.	2024	China	Parkinson’s disease (n=63) Controls (n=49)	Parkinson’s disease (27/36) Controls (28/21)	Parkinson’s disease (67.08 ± 7.87) Controls (68.65 ± 4.13)	Not reported	Cases: Hospital inpatients Controls: Hospital outpatients	PD was diagnosed using the Movement Disorders Society Criteria (Postuma et al., 2015). No other neurological histories other than PD; No Parkinson’s syndrome and multisystem atrophy; No severe anxiety, depression, schizophrenia, and other psychological diseases; No systemic diseases of the heart, liver, and kidney, diseases of the blood system, malignant tumors and other diseases that may affect cognitive function.	healthy volunteers	CD19+ B cells	6
10	Garfias S et al.	2022	Mexico	Parkinson’s disease (n=20) Controls (n=18)	Parkinson’s disease (10/10) Controls (7/10)	Parkinson’s disease (64.7 ± 9.5 ) Controls (67.5 ± 6.2)	2.5 ± 0.5	Cases: Not specified Controls: Not specified	PD patients met the UK Parkinson’s Disease Society Brain Bank criteria.age of onset was over 40 years for patients with PD. absence of infection, inflammatory diseases, cancer, and/or metabolic disorders; and not having taken anti-inflammatory treatment in the 3 months prior to inclusion.	absence of infection, inflammatory diseases, cancer, and/or metabolic disorders; and not having taken anti-inflammatory treatment in the 3 months prior to inclusion.	CD19+ B cells, regulatory B cells	6
11	Zhaoqi Yan et al.	2021	UK.Birmingham	Parkinson’s disease (n=41 Controls (n=40)	Parkinson’s disease (25/16) Controls (18/22)	Parkinson’s disease (62.95 ± 9.35 ) Controls (61.07 ± 10.19)	Not reported	Cases: Hospital outpatients Controls: Hospital outpatients	Diagnosis of PD by a fellowship-trained movement disorders specialist using UK Brain Bank criteria (at least 2 of the following: resting tremor, bradykinesia, and rigidity); male or female aged 30 years or older at the time of PD diagnosis; no more than 2 years since the initial diagnosis of PD and no more than 1 year on treatment for PD; and Hoehn and Yahr stage I or II. Without an atypical PD syndrome due to drugs, metabolic disorders, encephalitis, or degenerative diseases other than PD; No dementia; Not use of investigational drugs or devices within 60 days prior; known severe anemia (hematocrit <30); or a history of clinically significant autoimmune or inflammatory disorder.	HCs were age older than 30 years; no current diagnosis of PD or other neurodegenerative disorder; no history of PD in first-degree blood relatives; and a lack of positive responses on more than 3 items on a PD Screening Questionnaire. No severe anemia (hematocrit <30); No history of clinically significant autoimmune or inflammatory disorder; or use of immunomodulatory or anti-inflammatory drugs.	CD19+B cells, naïve B cells, SM B cells, USM B cells, DNM B cells	8
12	Zhuo Zhang et al.	2023	China	Early-stage Parkinson’s disease (n=23) Late-stage Parkinson’s disease(n=38) Controls (n=61)	Early-stage Parkinson’s disease(7/16) Late-stage Parkinson’s disease(21/17) Controls (28/33)	Early-stage Parkinson’s disease (61.04 ± 9.46 ) Late-stage Parkinson’s disease (65.53 ± 8.79 ) Controls (63.30 ± 9.09)	Early-stage Parkinson’s disease :2.00 ± 0.00 Late-stage Parkinson’s disease:3.00 ± 1.00	Cases: Hospital ipatients Controls: Hospital outpatients	Clinically established PD according to the Inter­national Parkinson and Movement Disorder Society (MDS) Clinical Diagnostic Criteria, and the ability to complete all clinical evaluations. No atypical parkinsonism or other CNS diseases, cancer, autoimmune disease or chronic infections, history of immunosuppressive treatment, receiving anti-inflammatory therapy, vaccination within the past six months, and cognitive impairment or clinically significant mood disorder preventing the patient from coop­ erating with the clinical evaluation.	None of the healthy participants received any vaccinations within the past six months or showed any signs of somatic disorders, such as cancer, autoimmune diseases, or acute/chronic inflammatory disorders.	CD19+B cells, naïve B cells, DNM B cells, USM memory B cells, SM B cells, Bregs, PBCs	7
13	Álvarez-Luquín DD et al.	2021	México	Parkinson’s disease (n=32) Controls (n=22)	Parkinson’s disease (18/14) Controls (12/10)	Parkinson’s disease (60.81 ± 10.23 ) Controls (55.59 ± 10.22)	Not reported	Cases: Hospital outpatients Controls: Hospital outpatients	patients were diagnosed with idiopathic PD. The patients were evaluated regularly by a neurologist, who adjusted the treatment as required for the disease to be in control according to international guidelines. PD patients treated with levodopa, pramipexole, or a levodopa/pramipexole combination were followed up (PD-1 yr and PD-2 yr). PD patients receiving any other treatment were excluded.	healthy subjects	CD19+ B cells, plasma cells, IL-10+ plasma cells (CD138+ IL-10+)	6
14	Álvarez-Luquín DD et al.	2019	México	Parkinson’s disease (n=32) Controls (n=22)	Parkinson’s disease (18/14) Controls (12/10)	Parkinson’s disease (60.81 ± 10.23 ) Controls (55.59 ± 10.22)	2.17 ± 0.88	Cases: Hospital outpatients Controls: Hospital outpatients	All diagnoses were performed by an expert neurologist at the INNN, following the United Kingdom Parkinson’s Disease Society Brain Bank (UK PDSBB) diagnosis criteria.	healthy subjects	CD19+ B cells, Bregs(CD19+ CD38hiCD24hi IL-10+), Functional Bregs(CD19- CD138+IL-10+)	6
15	Horvath S et al.	2015	USA	Parkinson’s disease (Caucasian(n=289,Hispanic(n=46) Controls (Caucasian(n=219,Hispanic(n=38)	Parkinson’s disease:Caucasian (164/125) Hispanic:(32/14) Controls: Caucasian (117/102) Hispanic:(18/20)	Not reported	Not reported	Cases: Community Controls: Community	Cases were identified with the help of local neurologists, clinics, and community outreach. Every PD patient was evaluated by a UCLA movement disorder specialist.	healthy subjects	plasma cells, B cells	5
16	Xiuzhen Zhao et al.	2020	China	Parkinson’s disease (n=39) Controls (n=26)	Parkinson’s disease (20/19) Controls (13/13)	Parkinson’s disease (61.49 ± 5.79 ) Controls (60.77 ± 6.77)	Not reported	Cases: Hospital inpatients Controls: Hospital outpatients	The disease was evaluated according to the clinical diagnostic criteria for PD put forth by the Movement Disorders Association (MDS) in 2015. No recent infection symptoms or suspected infection; No usage of any anti-inflammatory drugs (such as NSAIDs), hormones, and immunosuppressants in the past 3 months; No autoimmune diseases (such as systemic lupus erythematosus, rheumatoid arthritis, Sjogren’s syndrome, myasthenia gravis, multiple sclerosis, etc.); No severe digestive, circulatory, endocrine, and hematological disorders; and familial PD.	healthy controls	CD19+ B cells	6
17	Sun C et al.	2019	China	Parkinson’s disease (n=127) Controls (n=148)	Parkinson’s disease (75/52) Controls (84/64)	Parkinson’s disease (62.7 ± 12.5 ) Controls (60.3 ± 13.4)	Not reported	Cases: Hospital inpatients Controls: Hospital outpatients	All patients were diagnosed with clinically established PD or clinically probable PD according to the International Parkinson and Movement Disorder Society (MDS) Clinical Diagnostic Criteria. No alcoholism, cancer, autoimmune disease, or acute/chronic inflammatory disorders.	No alcoholism, cancer, autoimmune disease, or acute/chronic inflammatory disorders.	CD19+ B cells	7
18	Hurny A et al.	2013	Poland	Parkinson’s disease (n=24) Controls (n=29)	Parkinson’s disease (14/10) Controls (15/14)	Parkinson’s disease (67 ± 8.4 ) Controls (64.4 ± 9.4)	Not reported	Cases: Hospital inpatients Controls: Hospital outpatients	24 patients with early diagnosed PD have never before been treated with L-DOPA preparations. Peripheral blood was taken before the onset of treatment and after medication with L-DOPA compounds. The duration of treatment was 207 ± 12 days, ranging from 181 to 229 days. The patients were treated with 300 to 800 mg of an L-DOPA compound per day.	No evident disease of the central nervous system or extrapyramidal syndrome.	CD19+ B cells	7
19	Rocha NP et al.	2018	Brazil	Parkinson’s disease (n=40) Controls (n=25)	Parkinson’s disease (27/13) Controls (19/6)	Parkinson’s disease (68.71 ± 10.07 ) Controls (65.23 ± 8.75)	2.44 ± 0.69	Cases: Hospital outpatients Controls: Community	patients with PD diagnosed according to the UK PD Brain Bank criteria. No undergone previous neurosurgery or no other neurological disorder and/or cognitive decline (i.e., delirium or dementia), significant sensory impairment, and infectious or autoimmune diseases in activity in the previous 4 weeks. no use corticosteroids, antiinflammatories, or antibiotics in the 4 weeks prior to the study.	No undergone previous neurosurgery or no other neurological disorder and/or cognitive decline (i.e., delirium or dementia), significant sensory impairment, and infectious or autoimmune diseases in activity in the previous 4 weeks. no use corticosteroids, antiinflammatories, or antibiotics in the 4 weeks prior to the study.	CD19+ B cells	8
20	Perner C et al.	2019	USA	Parkinson’s disease (n=33) Controls (n=33)	Parkinson’s disease (16/17) Controls (20/13)	Parkinson’s disease (69 ± 10.4 ) Controls (63.7 ± 11.7)	3 ± 1	Cases: Hospital inpatients Controls: Hospital outpatients	PD patients were diagnosed according to the United Kingdom PD Society Brain Bank Diagnostic Criteria. No any inflammatory conditions (like diabetes, multiple sclerosis, autoimmune disease), cancer, and any current infections (as determined by clinical status, C-reactive protein, and blood leucocyte counts). No Parkinson’s dementia.	No any inflammatory conditions (like diabetes, multiple sclerosis, autoimmune disease), cancer, and any current infections (as determined by clinical status, C-reactive protein (CRP), and blood leucocyte counts). No Parkinson’s dementia.	CD19+ B cells	7

USM B cells, un-class-switched memory B cells; CSM B cells, Class-switched Memory B cells; DNM B cells, Double Negative Memory B cells; Bregs, regulatory B cells; PD, Parkinson’s disease; EPD, Early-stage Parkinson’s disease; LPD, Late-stage Parkinson’s disease; PBCs, Plasma blast cells; PBMCs, Peripheral blood mononuclear cells; HDs, Healthy donors; IL, Interleukin.

### 3.2 Methodological quality assessment

We used the NOS to assess the methodological quality of studies included in our systematic review. All 20 studies had an NOS score of ≥5, indicating a relatively high quality of these studies. The detailed quality assessment is presented in **Supplementary Table 3**.

### 3.3 Total B lymphocytes

The surface expression of CD19 and CD20 is induced early in B cell development in the bone marrow (26, 27). They are all expressed on subsets of B cells in the blood (28). Therefore, CD19 and CD20 are used to identify B cells (**Supplementary Table 2**). Researchers are increasingly intrigued by the potential role of B cells in the adaptive immune system’s contribution to the pathogenesis of PD. To understand the phenotypic and functional characteristics of circulating B cells in PD patients, sixteen studies (13–15, 21, 23, 29–39) reported the levels of CD19^+^ B cells in peripheral blood according to flow cytometry markers assessed. The results of eight studies (13–15, 31–33, 37, 38) showed a significant decrease in CD19^+^ B cells in PD patients compared with controls. Conversely, the results of other studies (21, 23, 29, 30, 34–36, 39) showed that there was no significant difference in CD19^+^ B cells between PD patients and controls. Moreover, two studies (22, 40) reported the alteration of CD20^+^ B cells in peripheral blood according to flow cytometry markers assessed, and the results showed that a significant decrease in CD20^+^ B cells in PD patients compared with controls. Furthermore, another study by Horvath et al. (41) reported the estimated proportions of B cells based on the Houseman method (42), and the results indicated that PD patients had fewer B cells. However, available information couldn’t be extracted. Therefore, we performed a qualitative analysis in our study. Result is presented in **Table 2**.

**Table 2:** Summary of clinical information and the results of B lymphocytes and B lymphocyte subsets in peripheral blood of PD patients.

Author	Immune cell types	Experimental methods	Disease stage(H&Y score)	Medication use	p-Value	Result
Claire H Stevens et al. (15)	CD19+ B cells	Flow cytometry	EPD	Levodopa(mg):300	P=0.004	In PD there were significant decreases in the numbers of CD19+ B cells compared to controls, and CD19+ B cells (p<0.0001) decreasing with age.
Jordi Bas et al. (29)	CD19+ B cells	Flow cytometry	Not reported	Thirty-four patients were under antiparkinso-nian treatment (levodopa in all cases, plus dopaminergic agonists in 19 cases, seven of which were also treated with selegiline) for a mean time of 5 ± 3.7 years (from 1 to 13) at the time of study.	P=0.002	There were significant decreases in the absolute values of CD19+ B cells compared to controls.
Fumitoshi Niwa et al. (40)	CD20+ B cells	Flow cytometry	EPD	LEDD (mg): 419.17 ± 237.14	P=0.022	The percentage of CD20+ cells was significantly lower in patients with PD than in the controls.
Kirsten M Scott et al. (14)	CD19+ B cells, transitional B cells, naïve B cells, CSM B cells, DNM B cells, USM B cells, plasma cells, regulatory B cells(CD1d+)	Flow cytometry	EPD	LEDD(mg):517.37 ± 367.66	P=0.04 (CD19+) P=0.017 (regulatory B cells)	The percentage of B lymphocytes is reduced overall in patients compared to controls, particularly in a subset enriched for regulatory B cells, and regulatory B cells are negatively correlated with disease severity. There were no differences between the patient/control groups in the proportions of B-cell subsets apart from a regulatory B cells in all Parkinson’s disease patients compared to matched controls.
Pingping Wang et al. (16)	CD19+ B cells, naïve B cells, USM B cells, CSM B cells, plasmablast/plasma cells	Flow cytometry, single cell RNA-seq	EPD	Levodopa and Benserazide Hydrochloride Tablets; Pramipexole Dihydrochloride Tablets; Amantadine Hydrochloride Tablets;	P=0.0027 (USM B cells) P=0.0047 (naïve B cells)	We compared the percentage of B cell subsets in PD patients and healthy controls. We observed significant increase of USM B cells and significant decrease of naïve B cells in PD patients.
Marina A. Gruden et al. (22)	CD20+ B cells	Flow cytometry	EPD	The L-DOPA dopamine precursor agents L-Dopa+carbidopa or L-Dopa+ benserazide, the non-ergot D2/D3 agonists Piribedil or pramipexole. The mean group dose for L-Dopa over 5.2± 0.7 treatment years was 411.5± 134.6 g/patient.	P<0.05	There were no statistically significant differences in the content of T and B lymphocytes in the blood sera of PD patients with a 5 and 10 year disease duration, though this does not exclude the possibility of divergence in later stages of the disease. In drug-treated PD patients, there were reduced counts of CD20 positive B-lympho-cytes (−16%).
Rui Li et al. (13)	CD19+ B cells, transitional B cells, USM B cells, CSM B cells, Naïve B cells, DNM B cells, plasma cells	Flow cytometry	Not reported	Discovery Cohort LEDD(mg):1110.5 ± 596.2 Validation Cohort LEDD(mg):416.7 ± 384.1	P<0.05 (B cells) P=0.001 (DNM B cells)	Absolute counts of circulating B cells are decreased in patients with PD relative to controls in the discovery cohort and confirmed in the validation cohort. We observed that among B-cell subsets, immature transitional B cells were disproportionally decreased in patients with PD. Within mature B cells, the numbers of DNM B cells were decreased in patients with PD, whereas no appreciable abnormalities were noted for other B-cell subsets.
Luan Cen et al. (21)	CD19+ B cells	Flow cytometry	EPD	Not reported	P=0.241	No significant difference was found for CD19+ B cells between the two groups. But the percentage of CD19+ B cells in male patients was lower than that in female patients (P = 0.021).
Xudong Zhao et al. (30)	CD19+ B cells	Flow cytometry	EPD/LPD	LED (mg):281.63 ± 62.80	p>0.05	There was no difference in B lymphocyte cells between the two groups.
Garfias S et al. (31)	CD19+ B cells, regulatory B cells	Flow cytometry	EPD	Not reported	P=0.008 (CD19+) P<0.001 (regulatory B cells (CD19+ CD5+ IL-10+) P<0.01 (CD19+ CD5+ FoxP3+) P<0.01 (CD19+ CD5+ IL-10+ compared to baseline) P<0.05 (CD19+compared to baseline);	We identified a significant decrease in the percentage of B cells. Significantly higher proportions of regulatory B (CD19+ CD5+ IL-10+ and CD19+ CD5+ FoxP3+)cells were detected in patients with PD than in controls. Compared to baseline, we also observed a decrease in the percentage of regulatory B cells (CD19+ CD5+ IL-10+), whereas the proportion of B cells (CD19+) increased.
Zhaoqi Yan et al. (23)	CD19+B cells, naïve B cells, SM B cells, USM B cells, DNM B cells	Flow cytometry	EPD	Not reported (no more than 1 year on treatment for PD)	P=0.011 (naïve B cells) P=0.032 (USM B cells) P=0.036 (DNM B cells)	Results indicated no significant difference of total CD19+ B cells(%) between patients with PD and controls. A significant decrease in naïve B cells, with a significant increase in both USM B cells and DNM B cells, was observed in patients with PD compared with controls, with no difference in SM B cells.
Zhuo Zhang et al. (32)	CD19+B cells, naïve B cells, DNM B cells, USM memory B cells, SM B cells, Bregs, PBCs	Flow cytometry	EPD (n=23) LPD (n=38)	LEDD(mg): EPD (375 ± 325) LPD (720 ± 443.13)	P=0.029 (CD19+) P < 0.001 (naïve B cells) P= 0.001 (Bregs) P= 0.010 (PBCs) P= 0.038 (DNM B cells)	The results of the comparison between groups indicated that compared with controls, the absolute counts of B lymphocytes were decreased in LPD patients.The results of comparison between groups showed that compared with controls, the percentage of naïve B cells was decreased in LPD patients, whereas those of Bregs, PBCs and DNM B cells were increased. Compared with controls, the percentage of Bregs was elevated in EPD patients (P= 0.006), with no significant difference in other B-lymphocyte subsets between the two groups.
Álvarez-Luquín DD et al. (33)	CD19+ B cells, plasma cells, IL-10+ plasma cells (CD138+ IL-10+)	Flow cytometry	EPD	Not reported	P = 0.048 (plasma cells) P = 0.0078 (IL-10+ plasma cells)	There was no significant difference in the total frequencies of CD19 cells (%) compared to the control group. we found a decrease in the number of plasma cells and the levels of IL-10+ plasma cells in the PD-2 year group with respect to PD-1 year.
Álvarez-Luquín DD et al. (34)	CD19+ B cells, Bregs(CD19+ CD38hiCD24hi IL-10+), Functional Bregs(CD19- CD138+IL-10+)	Flow cytometry	EPD	Patients with no previous dopaminergic treatment	P =0.930 (CD19+) P =0.049 (Functional Breg)	The analysis of regulatory B cell(%) subpopulations showed that the levels of functional Bregs were significantly lower in patients than in controls.
Horvath S et al. (41)	plasma cells, B cells	The epigenetic clock(based on the DNA methylation levels)	Not reported	Levodopa	P=0.00065 (plasma cells in Caucasians) P=1.6×10^–5^ (B cells in Caucasians) P=4.5×10^–5^ (B cells in Hispanics)	Compared to control samples, PD patients have more plasma cells, but fewer B cells.
Xiuzhen Zhao et al. (35)	CD19+ B cells	Flow cytometry	EPD	Levodopa(mg):300(150–400)	P= 0.014	The percentage of CD19+ B cells among PBMCs was significantly lower in PD patients than in controls. However, no significant difference was found between the early PD group and middle-advanced PD group.
Sun C et al. (36)	CD19+ B cells	Flow cytometry	Not reported	LEDD(mg): 617.3 ± 444.8	P>0.05	No obvious differences in CD19+ B cells (%) were detected between the PD and control groups.
Hurny A et al. (37)	CD19+ B cells	Flow cytometry	EPD	300 to 800 mg of an L-DOPA compound per day	P=0.004	Before treatment with L-DOPA, there was no significant difference in the percentage of CD19 lymphocytes compared to the control group. While the percentage of CD19 lymphocytes were decreased after treatment with L-DOPA.
Rocha NP et al. (38)	CD19+ B cells	Flow cytometry	EPD	Levodopa; Pramipexole; Entacapone; Amantadine;	P=0.41	Patients with PD and controls presented similar percentage of B lymphocytes (CD19+).
Perner C et al. (39)	CD19+ B cells	Flow cytometry	LPD	Not reported	P>0.05	The frequencies of CD19+ B cells(%) did not significantly differ between PD patients and HDs. ​

USM B cells, un-class-switched memory B cells; CSM B cells, Class-switched Memory B cells; DNM B cells, Double Negative Memory B cells; Bregs, regulatory B cells; PD, Parkinson’s disease; EPD, Early-stage Parkinson’s disease; LPD, Late-stage Parkinson’s disease; PBCs, Plasma blast cells; PBMCs, Peripheral blood mononuclear cells; HDs, Healthy donors; LEDD, Levodopa equivalent daily dose.

### 3.4 B lymphocyte subsets

B lymphocyte subsets were identified according to flow cytometry markers assessed (**Supplementary Table 2**).

#### 3.4.1 Transitional B cells

To elucidate the potential mechanisms underlying the roles of circulating B lymphocyte subsets in PD patients, two studies (13, 14) reported the levels of transitional B cells in peripheral of PD patients. The results of Rui Li et al. (13) indicated a decreased disproportionally in transitional B cells in PD patients compared with controls. However, the results of Kirsten M Scott et al. (14) indicated no significant difference in the levels of transitional B cells between PD patients and controls.

#### 3.4.2 Regulatory B cells

To elucidate the potential mechanisms underlying the roles of circulating B lymphocyte subsets in PD patients, four studies (14, 29, 32, 37) reported the alteration of regulatory B cells in peripheral blood of PD patients and controls. In the study by Kirsten M Scott et al., the results showed a reduction in CD1d ^+^ (regulatory) B cells in PD patients compared to controls. Similarly, Álvarez-Luquín et al. (29) also observed similar results that the levels of functional Bregs (CD19^+^ CD38 ^hi^ CD24 ^hi^ IL-10^+^) were significantly lower in PD patients than in controls. Meanwhile, they also reported other human regulatory B cells in PD patients and controls, including CD19^+^ IL-10^+^, Plasmatic cells IL-10^+^ (CD19^-^ CD138^+^ IL-10^+^), and regulatory B cells (CD19^+^ CD5^+^ CD1d^+^ FOXP3^+^ IL-10^+^), but the results indicated no significant difference between PD patients and controls. On the contrary, significantly higher proportions of regulatory B cells (CD19^+^ CD5^+^ IL-10^+^ and CD19^+^ CD5^+^ FoxP3^+^) were detected in PD patients than in controls reported by Garfias S et al (32). Similarly, We also observed that the percentage of regulatory B cells was elevated in early PD patients compared with controls reported by Zhuo Zhang et al.37 However, in their results, no significant difference was observed in the absolute counts of regulatory B cells between PD patients and controls.

#### 3.4.3 Naïve B cells

Five studies (13, 14, 16, 23, 37) reported the alteration of Naïve B cells in peripheral blood of PD patients and controls. Specifically, the results of three studies (16, 23, 37) indicated a significant decrease in naïve B cells in PD patients compared with controls. However, the results of two studies indicated no significant difference in naïve B cells between PD patients and controls (13, 14).

#### 3.4.4 Memory B cells

Memory B cells can be categorized into three groups: Un-class-switched Memory (USM) B cells, Class-switched Memory (CSM) B cells, and Double Negative Memory (DNM) B cells. Five studies (13, 14, 16, 23, 37) reported the levels of memory B cells in peripheral of PD patients. The results of two studies (16, 23) indicated a significant increase of USM B cells in PD patients compared with controls, while no statistically significant differences were observed in the other three studies (13, 14, 37). The alteration of CSM B cells in the peripheral blood of PD patients and controls was reported in five studies (13, 14, 16, 23, 37), but no statistically significant differences were observed. Four studies examined the alteration of DNM B cells in peripheral blood of PD patients and controls. The results of two studies (23, 37) indicated a significant increase in DNM B cells in PD patients. The results of Rui Li et al. (13) indicateda significant decrease in DNM B cells. However, the results of Kirsten M Scott et al (14) indicated no statistically significant differences in DNM B cells between PD patients and controls.

#### 3.4.5 Plasma cells

Six studies (13, 14, 16, 30, 37, 41) reported the alteration of plasma cells in peripheral blood of PD patients and controls. Notably, two studies found an increase in plasma cells among individuals with PD (37, 41). Conversely, Álvarez-Luquín DD et al. reported a decrease in plasma cells in PD patients (30). In the remaining three studies, no statistically significant differences were observed between PD patients and controls (13, 14, 16).

## 4 Discussion

Subtle changes in the central nervous system microenvironment caused by peripheral changes in blood circulation or other organs (functional impairment, ecological imbalance, or inflammation) may have a significant impact on the function of the central nervous system (43). Research has shown that IgG antibodies deposit on dopaminergic neurons in PD patients, and the Lewy bodies were also coated by IgG (5). Recent evidence suggests that B lymphocytes in peripheral blood may interact with the central nervous system in complex manners via the meningeal lymphatic system (17, 44, 45) and via the channels in the skull bone marrow that allow the egress of calvarial immune cells, including B lymphocytes (46). These findings suggest that B lymphocytes may participate in the inflammation of PD in complex manners, including centrally and peripherally (47). However, the alterations of B lymphocytes and B lymphocyte subsets in peripheral blood of PD patients remain unclear. Our systematic review showed that compared with the control group, there were changes in the levels of B lymphocytes and B lymphocyte subsets in the peripheral of PD patients, including total B lymphocytes, transitional B cells, regulatory B cells, naïve B cells, memory B cells, and plasma cells, which indicated that Parkinson’s disease may be associated with the immune dysfunction of B lymphocytes or B lymphocyte subsets in peripheral blood. To the best of our knowledge, this is the first systematic review to consider the immune phenotypes of peripheral blood cells in Parkinson’s disease patients. Our findings emphasize the potential role of adaptive immune dysfunction of B lymphocytes in the pathogenesis of Parkinson’s disease.

### 4.1 Total B lymphocytes

Firstly, we found an inconsistent result in the alteration of CD19^+^ B cells in the peripheral blood of PD patients among studies. Interestingly, we noticed a significant decrease in counts of CD19^+^ B cells in peripheral blood in PD patients compared to controls. However, the results of the studies showed no difference in the percentage of CD19^+^ B cells in peripheral blood between PD patients and controls. A possible explanation for this phenomenon could be that relative percentages are a holistic representation of the immune landscape at the time of sampling. Unlike the total cell count, changes in relative percentage of one cell type may be influenced by changes in another cell types (48). Therefore, according to the standard practice of immunology, we will focus on the results of the absolute counts of B cells in peripheral blood as primary. In our systematic review, the studies reviewed demonstrated that the alterations of CD19^+^ B cells in the peripheral blood are influenced by sex (49), race, age, disease severity, PD duration, and the impact of medication use. Clinical variability may be the reason for the differences between studies in the literature (**Supplementary Table 4**). So, it will be necessary to consider these factors further in future research.

A significant decrease in the level of B cells was observed in PD. Several studies have described possible reasons for the decrease in B lymphocytes in peripheral blood of PD patients, such as clinical severity (H&Y), disease progresses, disease duration, and α-synuclein pathology (**Supplementary Table 4**).

### 4.2 The role of B lymphocytes in the prognosis of PD

To investigate the impact of decreased B lymphocytes on the course of parkinson’s disease, Kirsten M Scott et al. (14) conducted a study comparing motor and histological outcomes in two groups of mice: mature B lymphocytes deficient μMT mice and mice treated with a CD20 monoclonal antibody that can deplete mature B cells. The results indicate that depleting B lymphocytes either genetically or using a monoclonal antibody targeting the B cell antigen CD20 can lead to worse motor outcomes and more extensive dopamine loss than controls. Their results indicated that B lymphocytes play an early protective role in dopaminergic cell loss. We have seen similar results in PD patients. The percentage of CD19^+^ B cells in PD patients with the scores on the Unified Parkinson’s Disease Rating Scale (UPDRS) >24 was significantly lower than that in patients with UPDRS score < 24 reported by Luan Cen et al. (21), which indicated that the level of CD19^+^ B cells was negatively correlated with PD severity. In another study, we also observed that the decrease in the number of CD19^+^ B cells varied with the clinical stage of PD (15). Overall, these findings demonstrate an association between changes in B lymphocytes and the progression of PD, indicating that alterations in B lymphocytes in peripheral blood could potentially serve as a predictor of PD progression.

### 4.3 B lymphocyte subsets

Neuroinflammation plays a fundamental role in the pathophysiology of PD. Neurohistological and neuroimaging studies support the presence of ongoing and end-stage neuroinflammatory processes in PD (50). Research has shown that the immune system is continuously activated in individuals with chronic inflammation, characterized by a deficiency in the number and function of these inhibitory cells in circulation (51). Bregs, an immunosuppressive cells, support immunological tolerance (52). By producing transforming growth factor β (TGF-β), interleukin-35 (IL-35), and interleukin-10 (IL-10), Bregs suppress immunopathology by inhibiting the expansion of pathogenic T cells and other proinflammatory lymphocytes (52). However, unlike regulatory T cells (Tregs), no unique biomarker or release factor was used to define Breg (53). Instead, the term Breg refers to B cells with regulatory functions. Transitional B cells and IL-10-producing B cells which are antiinflammatory B lymphocyte subsets are well recognized in the field of autoimmunity as important checkpoint regulators of autoreactive T cells (13, 54). In the studies we included, the results regarding peripheral Bregs in individuals with PD were inconsistent. In the results of Garfias S et al. (32), significantly higher proportions of Bregs were detected in PD patients than in controls. However, their results showed a decrease in the percentage of Bregs between baseline and follow-up, which indicated that the alterations of Bregs may be related to the duration of PD. Bregs dysfunction has been found in many autoimmune diseases (55–59), such as multiple sclerosis, pemphigus, systemic sclerosis, and myasthenia gravis, but further work is needed to determine the pathogenic significance and the mechanisms that underlie this.

Bregs have been associated with the inhibition of excessive inflammation (60) and play a crucial role in maintaining immune homeostasis (61). The use of transgenic mice lacking B cells (62), and more specifically IL-10-producing B cells (63), has shown that defective Breg cell development and function lead to chronic inflammation. In the study of Kirsten M Scott et al. (14), the evidence that B lymphocyte deficiency or depletion leads to worse pathological and behavioral outcomes was shown in a toxin-based mouse model of PD. Furthermore, Kirsten M Scott et al. reported that PD Patients who had a greater proportion of Bregs had better motor scores. This further demonstrates the important role of B lymphocytes and Bregs in the pathogenesis of PD. At the same time, this also indicates that B lymphocytes or Bregs can serve as targets for the treatment of PD. However, the role of B lymphocytes and Bregs remain unclear in PD patients. Their protective effect can be partially explained by alleviating various immune mediated inflammatory conditions.

In previous studies (64, 65), we noticed that the naïve B cell compartment was significantly reduced after the depletion of the B cell activating factor of the tumor necrosis factor family(BAFF). The BAFF plays critical roles in supporting the survival of mature naïve B cells (66, 67) and has a physiological role in B lymphocyte immune regulation (68). However, inappropriate generation of BAFF may be a key event that disrupts immune tolerance associated with systemic autoimmune diseases. Some evidence suggested that an increase in BAFF levels may not only directly interfere with B cell immunity by triggering their maturation and survival, but also indirectly interfere with B cell immunity by dysregulating innate immune responses (69) and T cell activation and balance (70). In turn, that can lead to the breakdown of immune tolerance, the production of autoantibodies, and sustained local intracerebral inflammation and tissue damage (68). In the study of Zhuo Zhang et al. (37), their results showed that the serum BAFF levels increased in PD patients. Tabalumab, fully anti-BAFF humanized monoclonal antibodies, has been tested in patients with relapsing-remitting multiple sclerosis in a phase II randomized, double-blind, placebo-controlled study (ClinicalTrials.gov study identifier: NCT00882999). The BAFF may also be potential targets for PD intervention. However, there are limited studies on the correlation between BAFF, peripheral blood B lymphocyte subsets, and the severity of PD have been retrieved. Therefore, the role of BAFF in PD remains unclear and warrants further research in the future.

Memory B cells can mediate rapid and strong antibody responses during secondary immune responses (28). Notably, a study provided evidence that the USM B cells exhibited faster and stronger re-stimulation potential and participated in early inflammatory response (71). In the study of Pingping Wang et al., single cell RNA and B cell receptor (BCR) sequencing for B cells from PD patients and controls were performed (16). Their results showed that the level of USM B cells (especially the USM2 subset) increased significantly in PD patients compared to controls, and the USM2 subset had strong activation characteristics and high proliferation potential. Pingping Wang et al. (16) speculate that the USM2 subset might be an important participant in the humoral immune response of PD and might ultimately promote the production of infiltrating antibodies in the brain of PD patients. On the other hand, the DNM B cells are believed to be associated with aging and senescence (72). Studies showed that in the peripheral blood of healthy individuals, the DNM B cells were detected at low levels (73) and were expanded in elderly patients (74) and Alzheimer’s disease (AD) patients (75). The DNM B cells are also associated with inflammatory response (23) and are thought to produce elevated levels of pro-inflammatory cytokines (72). In our article, the studies reviewed demonstrated inconsistent results in the alteration of DNM B cells in the peripheral blood of PD patients across studies. The accumulation of the DNM B cells in chronic infections, autoimmune diseases and with advanced age has been observed (76–78). However, the role of the DNM B cells is still unknown in PD patients.

B lymphocytes eventually differentiate into plasma cells and produce antibodies. Autoantibodies against α-synuclein, dopamine and melanin were found in the serum and cerebrospinal fluid of PD patients (79, 80), and the levels of α-synuclein autoantibodies in plasma and cerebrospinal fluid were associated with disease activity in patients with mild or moderate PD (81). Studies showed that antibody-based therapies against α-synuclein have achieved enviable results in clinical trials, and may be expected to provide a new treatment method for PD (82, 83). Memory B cells can differentiate into plasma cells in a secondary response, either through direct differentiation after antigen attack or by seeding of germinal centers that in turn generate new plasma cells (84). In the current research, we noticed an inconsistent result in the alteration of plasma cells in the peripheral blood of PD patients compared to controls. Due to the fact that plasma cells are not themselves circulating, the upstream progenitor cell pool must contribute (84). This may be the reason for the inconsistent results of plasma cell changes.

The alterations of B lymphocyte subsets are also associated with increased cytokine production in PD patients (13, 23). Although the evidence of cytokine abnormalities in peripheral blood has been well established in PD (85), the alterations of B lymphocyte subsets in this illness are relatively less understood. Identifying the immunophenotype of B lymphocyte subsets in PD may help elucidate the cellular sources of these known cytokine alterations. Future research on the cytokines as biomarkers and therapeutic targets for PD may be warranted (85).

### 4.4 The limitations of the studies reviewed

Immunological activation and neuroinflammation are pivotal in the pathogenesis of PD (86). Although these studies have reported the alterations of B lymphocytes and B lymphocyte subsets in peripheral blood of PD, most of them are cross-sectional from a study design standpoint. As a result, they can only capture a snapshot of the immune status of PD patients and cannot relate their findings to the course and outcomes of the patients (13), nor can they infer causality. Therefore, future studies that longitudinally assess the immune profiles of patients may provide more insights into the involvement of B lymphocytes and B lymphocyte subsets in the disease. Furthermore, the studies reviewed demonstrated that the alterations of B cells in the peripheral blood are subject to a variety of influences, including gender, race, age, disease severity, the duration of Parkinson’s disease, and medication use. Consequently, it is imperative that future research designs rigorously control for these confounding variables to mitigate their effects on the analysis of peripheral blood B cells.

### 4.5 Mechanism of B lymphocytes in PD

**Figure 2:**
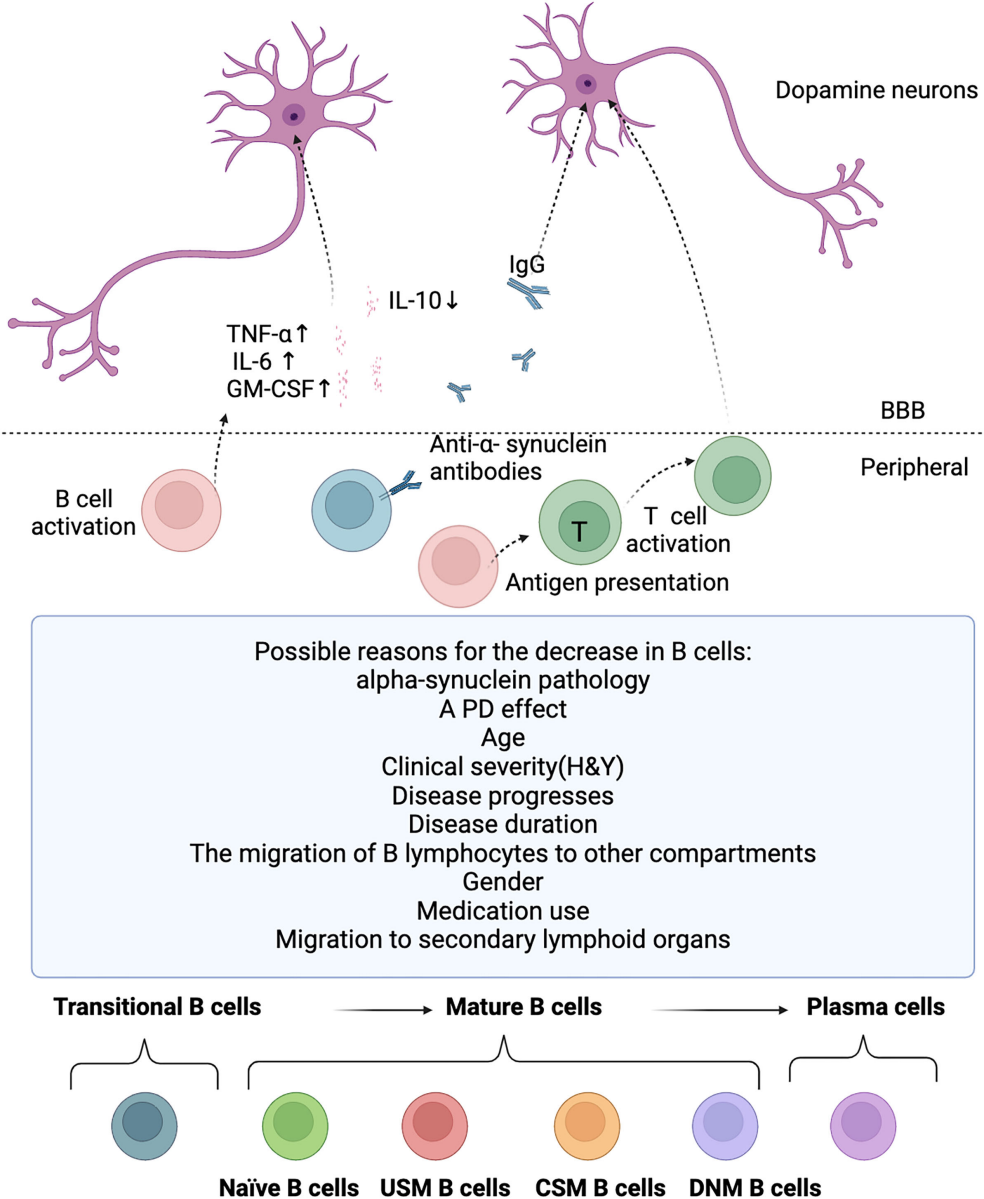
Potential roles for B cells in PD. The possible reasons for the decrease in B cells, the potential involvement of B cells in PD, and the diverse developmental stages of B cell subsets. B cell levels are reduced in PD patients for several possible reasons, including alpha-synuclein pathology, PD effect, age, clinical severity(H&Y), disease progresses, disease duration, the migration of B lymphocytes to other compartments, gender, medication use, and migration to secondary lymphoid organs. But B cells are activated and there is a significant increase in the pro-inflammatory cytokines TNF-α, IL-6 and GM-CSF, and a significant decrease in the anti-inflammatory cytokine IL-10 in PD, which eventually leads to deregulated neuroinflammation and subsequent dopaminergic neurodegeneration. IgG antibodies deposit on dopaminergic neurons in PD patients, and the Lewy bodies are also coated by IgG. Although anti-alpha-synuclein antibodies are present in the blood of PD patients, but it is still unclear whether these antibodies are the same as those that bind to neurons in the substantia nigra and Lewy bodies in these individuals. B cells are activated and the antigen presentation capacity of B cells is enhanced in PD patients. B lymphocytes can uptake, process, and present antigens to T cells, activating the immune response of T cells. T cell immune response can trigger type 1 pro-inflammatory activities and suppress type 2 anti-inflammatory activities, eventually resulting in deregulated neuroinflammation and subsequent dopaminergic neurodegeneration. T, T cell; USM B cells, un-class-switched memory B cells; CSM B cells, Class-switched Memory B cells; DNM B cells, Double Negative Memory B cells.

B lymphocytes are crucial for normal immune system development and its maintenance (17). In addition to producing antibodies, B lymphocytes also can release immune regulatory cytokines, antigen-presenting cell functions, and regulate T cells and the innate response (17, 18). B cells are activated and the antigen presentation capacity of B cells is enhanced in PD patients (16). It can uptake, process, and present antigens to T cells, activating the immune response of T cells. T cell immune response can trigger type 1 pro-inflammatory activities and suppress type 2 anti-inflammatory activities, eventually resulting in deregulated neuroinflammation and subsequent dopaminergic neurodegeneration (87). After the death of dopaminergic neurons, their antigens are presented to the immune system, with activation of B lymphocytes. Then, B lymphocytes or specific autoantibodies might enter the central nervous system through the dysfunctional blood brain barrier, produce cytokines that activate microglia, or release autoantibodies. This may lead to further inflammation and subsequent cell death (4). The possible mechanism of B cells in PD is shown in **Figure 2**.

### 4.6 Potential therapeutic target of PD

At present, no treatment method can slow down or arrest the progression of PD (88). The disease modification therapy that reduces the rate of neurodegeneration or prevents the progression of PD is still the biggest treatment requirement in PD (89), but informed by new insights into the immune dysfunction mechanism of PD in our study, immunotherapies targeting dysregulated B lymphocytes and B lymphocyte subsets (**Supplementary Table 4**) may be a promising strategy for disease-modifying potential.

### 4.7 Limitations

In the current research, there are several potential limitations: (1)The evolution of high-dimensional phenotype technology has identified many new B lymphocyte subsets (90–93). Given the marked diversity in B lymphocyte subsets phenotype, we selected the main subsets of B lymphocytes in peripheral blood for a systematic review; (2)It is crucial to adopt a unified and consistent method for identifying and describing B lymphocyte subsets to ensure the reproducibility of scientific research and and that similar conclusions can be drawn from them (28). However, to gain a more comprehensive understanding of the alteration in B lymphocytes and B lymphocyte subsets in peripheral blood of PD patients, in addition to flow cytometry, studies using other techniques [such as single-cell RNA-seq and the epigenetic clock (based on the DNA methylation levels)] to identify and describe the alteration in B lymphocytes and B lymphocyte subsets in peripheral blood of PD patients were also included in our study; (3)As is well known, it is difficult to infer causality from case-control studies. As a result of our research, it is currently unclear whether the observed alteration in B lymphocytes and B lymphocyte subsets in acquired immunity are a causal or secondary to central nervous system disorder associated with the pathogenesis of PD.

### 4.8 Conclusion

Our study comprehensively and systematically reviewed the alterations of B lymphocytes and B lymphocyte subsets and provided evidence of a decrease in B lymphocyte levels and immune dysfunction in B lymphocyte subsets in peripheral of PD patients. PD is associated with the alterations of B lymphocytes and (or) B lymphocyte subsets in peripheral blood. This study provides a novel perspective into the pathogenesis of PD, and future investigations into the B lymphocytes and (or) B lymphocyte subsets as biomarkers and therapeutic targets for PD may be warranted.

## Data Availability

The original contributions presented in the study are included in the article/**Supplementary Material**. Further inquiries can be directed to the corresponding author.
