# Supplementary material for: Aberrations in peripheral B lymphocytes and B lymphocyte subsets levels in Parkinson disease: a systematic review
Source: Front Immunol. 2025 Mar 31;16:1526095. doi: 10.3389/fimmu.2025.1526095 (PMC11994702; doi:10.3389/fimmu.2025.1526095)
Supplement: Supplementary file 1 [file Table1.docx]

**The Search Strategy on PubMed（1465）**

#1 (Parkinson Disease [Mesh] ) OR Idiopathic Parkinson's Disease OR Lewy Body Parkinson's Disease OR Parkinson's Disease, Idiopathic OR Parkinson's Disease, Lewy Body OR Parkinson Disease, Idiopathic OR Parkinson's Disease OR Idiopathic Parkinson Disease OR Lewy Body Parkinson Disease OR Primary Parkinsonism OR Parkinsonism, Primary OR Paralysis Agitans

#2 (B-Lymphocytes [Mesh] ) OR B Lymphocytes OR Bursa-Dependent Lymphocytes OR Bursa Dependent Lymphocytes OR Bursa-Dependent Lymphocyte OR Lymphocyte, Bursa-Dependent OR Lymphocytes, Bursa-Dependent OR B-Cells, Lymphocyte OR B Cells, Lymphocyte OR B-Cell, Lymphocyte OR Lymphocyte B-Cell OR Lymphocyte B-Cells OR B-Lymphocyte OR B Lymphocyte OR CD19^+^ B cells OR CD20^+^ B cells

#3 **(**Precursor Cells, B-Lymphoid [Mesh] ) OR Immature B Cell OR B Cell, Immature OR B Cells, Immature OR Cell, Immature B OR Cells, Immature B OR Immature B Cells OR Immature B-Lymphocyte OR B-Lymphocyte, Immature OR B-Lymphocytes, Immature OR Immature B Lymphocyte OR Transitional B-Lymphocyte OR B-Lymphocyte, Transitional OR B-Lymphocytes, Transitional OR Transitional B Lymphocyte OR Transitional B-Lymphocytes OR Transitional B Lymphocytes OR Immature B-Lymphocytes OR Immature B Lymphocytes OR Immature B-Cells OR Transitional B-Cells OR Transitional B Cells OR Immature B-Cell OR B-Cell, Immature OR B-Cells, Immature OR Transitional B-Cell OR B-Cell, Transitional OR B-Cells, Transitional OR Transitional B Cell OR Cell, B-Lymphoid Precursor OR Cells, B-Lymphoid Precursor OR Precursor Cell, B-Lymphoid OR Precursor Cells, B Lymphoid OR B-Lymphoid Precursor Cell OR B Lymphoid Precursor Cell OR B-Lymphoid Precursor Cells OR B Lymphoid Precursor Cells OR Pre-B Lymphocytes OR Pre B Lymphocytes OR Pre-B-Cells OR Precursor B-Cells OR Precursor B Cells OR Precursor B-Lymphocyte OR B-Lymphocyte, Precursor OR B-Lymphocytes, Precursor OR Precursor B Lymphocyte OR Pre-B Lymphocyte OR Lymphocyte, Pre-B OR Lymphocytes, Pre-B OR Pre B Lymphocyte OR Pre-B Cell OR Cell, Pre-B OR Cells, Pre-B OR Pre B Cell OR Pre-B-Cell OR Precursor B-Cell OR B-Cell, Precursor OR B-Cells, Precursor OR Precursor B Cell OR Pre-B Cells OR Pre B Cells OR Precursor B-Lymphocytes OR Precursor B Lymphocytes OR Pro-B Lymphocytes OR Lymphocyte, Pro-B OR Lymphocytes, Pro-B OR Pro B Lymphocytes OR Progenitor B-Cell OR B-Cell, Progenitor OR B-Cells, Progenitor OR Progenitor B Cell OR Pro-B-Cells OR Progenitor B-Cells OR Progenitor B Cells OR Pro-B Cel OR Pro B Cell OR Pro-B-Cell OR Pro-B Cells OR Cell, Pro-B OR Cells, Pro-B OR Pro B Cells OR Pro-B Lymphocyte OR Pro B Lymphocyte OR Progenitor B-Lymphocyte OR B-Lymphocyte, Progenitor OR B-Lymphocytes, Progenitor OR Progenitor B Lymphocyte OR Progenitor B-Lymphocytes OR Progenitor B Lymphocytes

#4 **(**B-Lymphocytes, Regulatory [Mesh] ) OR B Lymphocytes, Regulatory OR B-Lymphocyte, Regulatory OR Regulatory B-Lymphocyte OR Regulatory B-Lymphocytes OR Breg Cell OR Cell, Breg OR Cells, Breg OR Regulatory B-Cells OR B-Cells, Regulatory OR B-Cell, Regulatory OR Regulatory B-Cell OR Breg Cells OR Regulatory B Cell OR B Cell, Regulatory OR B Cells, Regulatory OR Cell, Regulatory B OR Cells, Regulatory B OR Regulatory B Cells

#5 naïve B cells OR naive B cells

#6 **(**Memory B cells [Mesh] ) OR B Cell, Memory OR Memory B Cell OR Memory B-Lymphocytes OR B-Lymphocyte, Memory OR B-Lymphocytes, Memory OR Memory B Lymphocytes OR Memory B-Lymphocyte

#7 **(**Plasma cells [Mesh] ) OR Cell, Plasma OR Cells, Plasma OR Plasma Cell OR Plasmacytes OR Plasmacyte

#8 #2 OR #3 OR #4 OR #5 OR #6 OR #7

#9 #1 AND #8

**The Search Strategy on Cochrane Library（141）**

Limits: Clinical trials

#1 MeSH descriptor: [Parkinson Disease] explode all trees 5983

#2 (Parkinson Disease or Idiopathic Parkinson's Disease or Lewy Body Parkinson's Disease or Parkinson's Disease, Idiopathic or Parkinson's Disease, Lewy Body or Parkinson Disease, Idiopathic or Parkinson's Disease or Idiopathic Parkinson Disease or Lewy Body Parkinson Disease or Primary Parkinsonism or Parkinsonism, Primary or Paralysis Agitans):ti,ab,kw (Word variations have been searched) 13263

#3 MeSH descriptor: [B-Lymphocytes] explode all trees 733

#4 (Lymphocytes or B Lymphocytes or Bursa-Dependent Lymphocytes or Bursa Dependent Lymphocytes or Bursa-Dependent Lymphocyte or Lymphocyte, Bursa-Dependent or Lymphocytes, Bursa-Dependent or B-Cells, Lymphocyte or B Cells, Lymphocyte or B-Cell, Lymphocyte or Lymphocyte B-Cell or Lymphocyte B-Cells or B-Lymphocyte or B Lymphocyte):ti,ab,kw (Word variations have been searched) 27668

#5 MeSH descriptor: [Precursor Cells, B-Lymphoid] explode all trees 6

#6 (Precursor Cells, B-Lymphoid or Cell, B-Lymphoid Precursor or Cells, B-Lymphoid Precursor or Precursor Cell, B-Lymphoid or Precursor Cells, B Lymphoid or Immature B Cell or B Cell, Immature or B Cells, Immature or Cell, Immature B or Cells, Immature B or Immature B Cells or B-Lymphoid Precursor Cell or B Lymphoid Precursor Cell or Immature B-Lymphocyte or B-Lymphocyte, Immature or B-Lymphocytes, Immature or Immature B Lymphocyte or B-Lymphoid Precursor Cells or B Lymphoid Precursor Cells or Transitional B-Lymphocyte or B-Lymphocyte, Transitional or B-Lymphocytes, Transitional or Transitional B Lymphocyte or Transitional B-Lymphocytes or Transitional B Lymphocytes or Immature B-Lymphocytes or Immature B Lymphocytes or Immature B-Cells or Transitional B-Cells or Transitional B Cells or Immature B-Cell or B-Cell, Immature or B-Cells, Immature or Transitional B-Cell or B-Cell, Transitional or B-Cells, Transitional or Transitional B Cell or Pre-B Lymphocytes or Pre B Lymphocytes or Pre-B-Cells or Precursor B-Cells or Precursor B Cells or Precursor B-Lymphocyte or B-Lymphocyte, Precursor or B-Lymphocytes, Precursor or Precursor B Lymphocyte or Pre-B Lymphocyte or Lymphocyte, Pre-B or Lymphocytes, Pre-B or Pre B Lymphocyte or Pre-B Cell or Cell, Pre-B or Cells, Pre-B or Pre B Cell or Pre-B-Cell or Precursor B-Cell or B-Cell, Precursor or B-Cells, Precursor or Precursor B Cell or Pre-B Cells or Pre B Cells or Precursor B-Lymphocytes or Precursor B Lymphocytes or Pro-B Lymphocytes or Lymphocyte, Pro-B or Lymphocytes, Pro-B or Pro B Lymphocytes or Progenitor B-Cell or B-Cell, Progenitor or B-Cells, Progenitor or Progenitor B Cell or Pro-B-Cells or Progenitor B-Cells or Progenitor B Cells or Pro-B Cell or Pro B Cell or Pro-B-Cell or Pro-B Cells or Cell, Pro-B or Cells, Pro-B or Pro B Cells or Pro-B Lymphocyte or Pro B Lymphocyte or Progenitor B-Lymphocyte or B-Lymphocyte, Progenitor or B-Lymphocytes, Progenitor or Progenitor B Lymphocyte or Progenitor B-Lymphocytes or Progenitor B Lymphocytes):ti,ab,kw (Word variations have been searched) 5130

#7 MeSH descriptor: [B-Lymphocytes, Regulatory] explode all trees 5

#8 (Lymphocytes, Regulatory or B Lymphocytes, Regulatory or B-Lymphocyte, Regulatory or Regulatory B-Lymphocyte or Regulatory B-Lymphocytes or Breg Cell or Cell, Breg or Cells, Breg or Regulatory B-Cells or B-Cells, Regulatory or B-Cell, Regulatory or Regulatory B-Cell or Breg Cells or Regulatory B Cell or B Cell, Regulatory or B Cells, Regulatory or Cell, Regulatory B or Cells, Regulatory B or Regulatory B Cells):ti,ab,kw (Word variations have been searched) 2247

#9 (naïve B cells or naive B cells):ti,ab,kw (Word variations have been searched) 1525

#10 MeSH descriptor: [Memory B Cells] explode all trees 3

#11 (Memory B cells or B Cell, Memory or Memory B Cell or Memory B-Lymphocytes or B-Lymphocyte, Memory or B-Lymphocytes, Memory or Memory B Lymphocytes or Memory B-Lymphocyte):ti,ab,kw (Word variations have been searched) 934

#12 MeSH descriptor: [Plasma Cells] explode all trees 53

#13 (Plasma cells or Cell, Plasma or Cells, Plasma or Plasma Cell or Plasmacytes or Plasmacyte):ti,ab,kw (Word variations have been searched) 18315

#14 #1 or #2 13263

#15 #3 or #4 27711

#16 #5 or #6 5130

#17 #7 or #8 2247

#18 #10 or #11 934

#19 #12 or #13 18315

#20 #14 and (#15 or #16 or #17 or #18 or #19 or #9) 141

**The Search Strategy on MEDLINE（1657）**

#1 TS=(Parkinson Disease) OR AB=(Idiopathic Parkinson's Disease OR Lewy Body Parkinson's Disease OR Parkinson's Disease, Idiopathic OR Parkinson's Disease, Lewy Body OR Parkinson Disease, Idiopathic OR Parkinson's Disease OR Idiopathic Parkinson Disease OR Lewy Body Parkinson Disease OR Primary Parkinsonism OR Parkinsonism, Primary OR Paralysis Agitans)

#2 TS=(B-Lymphocytes) OR AB=(B Lymphocytes OR Bursa-Dependent Lymphocytes OR Bursa Dependent Lymphocytes OR Bursa-Dependent Lymphocyte OR Lymphocyte, Bursa-Dependent OR Lymphocytes, Bursa-Dependent OR B-Cells, Lymphocyte OR B Cells, Lymphocyte OR B-Cell, Lymphocyte OR Lymphocyte B-Cell OR Lymphocyte B-Cells OR B-Lymphocyte OR B Lymphocyte OR CD19+ B cells OR CD20+ B cells)

#3 TS=(Precursor Cells, B-Lymphoid) OR AB=(Transitional B cells OR Immature B Cell OR B Cell, Immature OR B Cells, Immature OR Cell, Immature B OR Cells, Immature B OR Immature B Cells OR Immature B-Lymphocyte OR B-Lymphocyte, Immature OR B-Lymphocytes, Immature OR Immature B Lymphocyte OR Transitional B-Lymphocyte OR B-Lymphocyte, Transitional OR B-Lymphocytes, Transitional OR Transitional B Lymphocyte OR Transitional B-Lymphocytes OR Transitional B Lymphocytes OR Immature B-Lymphocytes OR Immature B Lymphocytes OR Immature B-Cells OR Transitional B-Cells OR Transitional B Cells OR Immature B-Cell OR B-Cell, Immature OR B-Cells, Immature OR Transitional B-Cell OR B-Cell, Transitional OR B-Cells, Transitional OR Transitional B Cell OR Cell, B-Lymphoid Precursor OR Cells, B-Lymphoid Precursor OR Precursor Cell, B-Lymphoid OR Precursor Cells, B Lymphoid OR B-Lymphoid Precursor Cell OR B Lymphoid Precursor Cell OR B-Lymphoid Precursor Cells OR B Lymphoid Precursor Cells OR Pre-B Lymphocytes OR Pre B Lymphocytes OR Pre-B-Cells OR Precursor B-Cells OR Precursor B Cells OR Precursor B-Lymphocyte OR B-Lymphocyte, Precursor OR B-Lymphocytes, Precursor OR Precursor B Lymphocyte OR Pre-B Lymphocyte OR Lymphocyte, Pre-B OR Lymphocytes, Pre-B OR Pre B Lymphocyte OR Pre-B Cell OR Cell, Pre-B OR Cells, Pre-B OR Pre B Cell OR Pre-B-Cell OR Precursor B-Cell OR B-Cell, Precursor OR B-Cells, Precursor OR Precursor B Cell OR Pre-B Cells OR Pre B Cells OR Precursor B-Lymphocytes OR Precursor B Lymphocytes OR Pro-B Lymphocytes OR Lymphocyte, Pro-B OR Lymphocytes, Pro-B OR Pro B Lymphocytes OR Progenitor B-Cell OR B-Cell, Progenitor OR B-Cells, Progenitor OR Progenitor B Cell OR Pro-B-Cells OR Progenitor B-Cells OR Progenitor B Cells OR Pro-B Cel OR Pro B Cell OR Pro-B-Cell OR Pro-B Cells OR Cell, Pro-B OR Cells, Pro-B OR Pro B Cells OR Pro-B Lymphocyte OR Pro B Lymphocyte OR Progenitor B-Lymphocyte OR B-Lymphocyte, Progenitor OR B-Lymphocytes, Progenitor OR Progenitor B Lymphocyte OR Progenitor B-Lymphocytes OR Progenitor B Lymphocytes)

#4 TS=(Regulatory B cells) OR AB=(B Lymphocytes, Regulatory OR B-Lymphocyte, Regulatory OR Regulatory B-Lymphocyte OR Regulatory B-Lymphocytes OR Breg Cell OR Cell, Breg OR Cells, Breg OR Regulatory B-Cells OR B-Cells, Regulatory OR B-Cell, Regulatory OR Regulatory B-Cell OR Breg Cells OR Regulatory B Cell OR B Cell, Regulatory OR B Cells, Regulatory OR Cell, Regulatory B OR Cells, Regulatory B OR Regulatory B Cells)

#5 AB=(naïve B cells OR naive B cells)

#6 TS=(Memory B cells) OR AB=(B Cell, Memory OR Memory B Cell OR Memory B-Lymphocytes OR B-Lymphocyte, Memory OR B-Lymphocytes, Memory OR Memory B Lymphocytes OR Memory B-Lymphocyte)

#7 TS=(Plasma cells) OR AB=(Cell, Plasma OR Cells, Plasma OR Plasma Cell OR Plasmacytes OR Plasmacyte)

#8 #2 OR #3 OR #4 OR #5 OR #6 OR #7

#9 #1 AND #8
