# Supplementary material for: Aberrations in peripheral B lymphocytes and B lymphocyte subsets levels in Parkinson disease: a systematic review
Source: Front Immunol. 2025 Mar 31;16:1526095. doi: 10.3389/fimmu.2025.1526095 (PMC11994702; doi:10.3389/fimmu.2025.1526095)
Supplement: Supplementary file 3 [file Table3.docx]

| **Cell Class** | | **Individual Cell Types** |
| --- | --- | --- |
| B lymphocytes | B cells | B cells  B Lymphocytes  CD19+  CD20+ |
|  | Transitional B cells | transitional B cells  CD27−CD24^HI^CD38^HI^  CD27−CD24^int^CD38^int^ |
|  | Regulatory B cells | Regulatory B cells  Bregs  CD1d ^+^  CD19^+^ CD38 ^hi^ CD24 ^hi^ IL-10^+^  CD19^+^ IL-10^+^  CD19^-^ CD138^+^ IL-10^+^  CD19^+^ CD5^+^ CD1d ^+^ FOXP3^+^ IL-10^+^  CD19^+^ CD5^+^ IL-10^+^  CD19^+^ CD5^+^ FoxP3^+^  CD19^+^CD24^hi^CD38^hi^ |
|  | Naïve B cells | Naïve B cells  Naive B cells  CD19^+^CD27^–^IgD^+^  CD27^–^IgD^+^ |
|  | Memory B cells | Un-class-switched Memory B cells  Class-switched Memory B cells  Double Negative Memory B cells  CD27^−^ IgD^−^  CD27^+^ IgD^+^  CD27^+^ IgD^−^  CD19^+^CD27^–^IgD^–^  CD19^+^CD27^+^IgD^+^  CD19^+^CD27^+^IgD^–^ |
|  | Plasma cells | plasma cells  Plasmablasts  CD138+  CD38+ CD138+  CD19+CD24–CD38+ |
