# Supplementary material for: Aberrations in peripheral B lymphocytes and B lymphocyte subsets levels in Parkinson disease: a systematic review
Source: Front Immunol. 2025 Mar 31;16:1526095. doi: 10.3389/fimmu.2025.1526095 (PMC11994702; doi:10.3389/fimmu.2025.1526095)
Supplement: Supplementary file 4 [file Table4.docx]

| **No.** | **Authors (year)** | **Selection** | | | | **Comparability** | **Exposure** | | **NOS quality score**  **(max 8)** |
| --- | --- | --- | --- | --- | --- | --- | --- | --- | --- |
|  |  | **Is case definition adequate?** | **Representativeness of cases** | **Selection of controls** | **Definition of controls** | **Comparability of cohorts on basis of design or analysis controlled for confounders** | **Ascertainment of exposure** | **Same method of ascertainment for cases and controls** |  |
| 1 | Claire H Stevens et al. (2012) | 1 | 1 | 0 | 1 | 1 | 1 | 1 | 6 |
| 2 | Jordi Bas et al.  (2001) | 1 | 1 | 1 | 0 | 1 | 1 | 1 | 6 |
| 3 | Fumitoshi Niwa et al. (2011) | 1 | 1 | 1 | 1 | 1 | 1 | 1 | 7 |
| 4 | Kirsten M Scott et al. (2023) | 1 | 1 | 1 | 1 | 2 | 1 | 1 | 8 |
| 5 | Pingping Wang et al. (2022) | 0 | 1 | 1 | 1 | 1 | 1 | 1 | 6 |
| 6 | Marina A. Gruden et al. (2011) | 1 | 1 | 0 | 1 | 1 | 1 | 1 | 6 |
| 7 | Rui Li et al. (2022) | 1 | 1 | 1 | 1 | 1 | 1 | 1 | 7 |
| 8 | Luan Cen et al. (2017) | 1 | 1 | 1 | 1 | 1 | 1 | 1 | 7 |
| 9 | Xudong Zhao et al. (2023) | 1 | 1 | 1 | 0 | 1 | 1 | 1 | 6 |
| 10 | Garfias S et al. (2019) | 1 | 1 | 0 | 1 | 1 | 1 | 1 | 6 |
| 11 | Zhaoqi Yan et al. (2021) | 1 | 1 | 1 | 1 | 2 | 1 | 1 | 8 |
| 12 | Zhuo Zhang et al. (2023) | 1 | 1 | 1 | 1 | 1 | 1 | 1 | 7 |
| 13 | Álvarez-Luquín DD et al(2021). | 1 | 1 | 1 | 0 | 1 | 1 | 1 | 6 |
| 14 | Álvarez-Luquín DD et al(2019). | 1 | 1 | 1 | 0 | 1 | 1 | 1 | 6 |
| 15 | Horvath S et al. (2015) | 1 | 1 | 1 | 0 | 0 | 1 | 1 | 5 |
| 16 | Xiuzhen Zhao et al. (2020) | 1 | 1 | 1 | 0 | 1 | 1 | 1 | 6 |
| 17 | Sun C et al. (2019) | 1 | 1 | 1 | 1 | 1 | 1 | 1 | 7 |
| 18 | Hurny A et al. (2013) | 1 | 1 | 1 | 1 | 1 | 1 | 1 | 7 |
| 19 | Rocha NP et al. (2018) | 1 | 1 | 1 | 1 | 2 | 1 | 1 | 8 |
| 20 | Perner C et al. (2019) | 1 | 1 | 1 | 1 | 1 | 1 | 1 | 7 |
