# Supplementary material for: Aberrations in peripheral B lymphocytes and B lymphocyte subsets levels in Parkinson disease: a systematic review
Source: Front Immunol. 2025 Mar 31;16:1526095. doi: 10.3389/fimmu.2025.1526095 (PMC11994702; doi:10.3389/fimmu.2025.1526095)
Supplement: Supplementary file 5 [file Table5.docx]

| **Analysis of Differential Results** | | | | |
| --- | --- | --- | --- | --- |
| Clinical variability | **Author** | **Result** | | **Possible influencing factors** |
|  | Luan Cen et al.[27] | They reported that there was no difference in CD19^+^ B cells between PD patients and controls, while the percentage of CD19^+^ B cells in male patients was lower than that in female patients. And they found that the percentage of CD19^+^ B cells in those with a score ＞24 was lower than in those with a score ＜24. | | Gender  PD Severity |
|  | Hurny A et al.[29] | Before treatment with L-DOPA, there was no significant difference in the percentage of CD19 lymphocytes compared to the control group. While the percentage of CD19 lymphocytes were decreased after treatment with L-DOPA. | | Medication use |
|  | Kedmi M et al.[48] | They found decreased expression of B cell-related genes in leukocytes of Ashkenazi women with PD, and their results suggested that the downregulation of genes related to B cell activity reflects the involvement of these cells in PD and represents a gender-specific molecular aspect in PD. | | Gender  Race |
|  | Álvarez-Luquín DD et al.[25] | There was no significant difference in the total frequencies of CD19 cells (%) compared to the control group. They found a decrease in the number of plasma cells in the PD-2 yr group with respect to PD-1 yr; interestingly, they found a positive correlation with age in the in PD-0 yr group. Additionally, they found a decrease in the levels of IL-10^+^ plasma cells in the PD-2 yr group with respect to PD-1 yr. On the other hand, they found a negative correlation between IL-10^+^ plasma cells with the H&Y scale score at PD-1 yr in patients treated with levodopa in combination with pramipexole. Also, decreased levels of lip-AP B cells were observed in the PD-1 yr and PD-2 yr groups with respect to PD-0 yr, while no significant changes were observed in the levels of activated B cells between times. Nevertheless, when the patients were stratified by treatment type, we observed that patients treated with a levodopa/pramipexole combination showed decreased levels of IL-10^+^ plasma cells after 2 years of treatment. | | Age  PD Severity  PD duration  Medication use |
| Testing methods | Rocha NP et al.[31] | Patients with PD and controls presented similar percentage of B lymphocytes (CD19^+^). | | Presentation method of results |
|  | Sun C et al.[32] | No obvious differences in CD19^+^ B cells (%) were detected between the PD and control groups. But the number of people with CD19^+^ B cells deviating from the normal range in the PD group was clearly higher than that in the control group. | | Presentation method of results |
|  | Álvarez-Luquín DD et al.[24] | Results indicated no significant difference of total CD19^+^ B cells(%) between patients with PD and controls. The analysis of regulatory B cell(%) subpopulations showed that the levels of functional Bregs were significantly lower in patients than in controls. | | Presentation method of results |
|  | Zhaoqi Yan et al.[33] | Results indicated no significant difference of total CD19^+^ B cells(%) between patients with PD and controls. A significant decrease in naïve B cells, with a significant increase in both USM B cells and DNM B cells, was observed in patients with PD compared with controls, with no difference in SM B cells. | | Presentation method of results |
|  | Perner C et al.[30] | The frequencies of CD19^+^ B cells(%) did not significantly differ between PD patients and HDs. | | Presentation method of results |
| **Possible reasons for the decrease in B cells** | | | | |
|  | **Author** | **Result** | | **Possible influencing factors** |
|  | Kirsten M Scott et al.[14] | They suggested that the decrease in the number of circulating B lymphocytes was not clearly driven by a decrease in any one subset, but by a global reduction in B lymphocytes. Additionally, they proposed that the decrease in the number of circulating B lymphocytes was associated with alpha-synuclein pathology. | | A global reduction in B cells  and alpha-synuclein pathology |
|  | Claire H Stevens et al.[15] | The authors suggested that the ongoing decrease of B lymphocytes in the peripheral blood of PD patients is a PD effect.  The number of CD19^+^ B cells decreasing with age.  H&Y stage negatively correlated with CD19^+^ cells;  Their results suggest that as the disease progresses there is an ongoing loss of CD19^+^ B cells.  A reduction in the numbers of total CD19^+^ cells was observed with increasing disease duration.  The assessment of eight PD patients before and after the onset of levodopa treatment showed a significant reduction in the numbers  of CD19^+^ cells post commencement of medication. | | A PD effect  Age  Clinical severity(H&Y)  Disease progresses  Disease duration  Levodopa treatment |
|  | Scott, K. M et al.[43] | The authors believed that the reduction in peripheral B lymphocyte numbers may also represent the migration of B lymphocytes to other compartments, such as cerebrospinal fluid or meninges, or represent the result of chronic stimulation. | | The migration of B lymphocytes to other compartments |
|  | Luan Cen et al.[27] | The percentage of CD19^+^ B cells in male patients was lower than that in female patients. And they found that the percentage of CD19^+^ B cells in those with a score ＞24 was lower than in those with a score ＜24. | | Gender  PD Severity |
|  | Marina A. Gruden et al.[38] | There were no statistically significant differences in the content of B lymphocytes in the blood sera of PD patients with a 5- and 10-year disease duration, though this does not exclude the possibility of divergence in later stages of the disease.In drug-treated PD patients there were reduced counts of CD20 positive B-lymphocytes (−16%). | | Medication use |
|  | Zhaoqi Yan et al.[33] | Whether this decrease in circulating CXCR3+ memory B cells in patients with PD reflects enhanced migration to secondary lymphoid organs is an intriguing possibility. | | Migration to secondary lymphoid organs |
|  | Hurny A et al.[29] | After treatment with L-DOPA, the percentage of CD19 lymphocytes was decreased. | | Medication use |
| **Potential therapeutic target analysis** | | | | |
|  | **Author** | | **Result** | **Potential therapeutic target** |
|  | Luan Cen et al.[27]  Kirsten M Scott et al.[14] | | A significant decrease in the level of B cells was observed in PD. B lymphocyte deficiency or depletion can lead to worse motor outcomes and more extensive dopamine loss than controls. | B lymphocyte |
|  | Kirsten M Scott et al.[14]  Álvarez-Luquín et al.[24]  Garfias S et al.[28]  Zhuo Zhang et al.[34] | | Bregs have been associated with the inhibition of excessive inflammation and play a crucial role in maintaining immune homeostasis; however, the results regarding peripheral Bregs in individuals with PD have been inconsistent. Dysfunction of Bregs has been observed in PD. | Bregs |
|  | Pingping Wang et al.[16]  Zhaoqi Yan et al.[33]  Zhuo Zhang et al.[34] | | A significant decrease in naïve B cells in PD patients compared with controls. The BAFF plays critical roles in supporting the survival of mature naïve B cells and has a physiological role in B lymphocyte immune regulation.. Elevated serum BAFF levels were observed in PD patients. Increase in BAFF levels may not only directly interfere with B cell immunity by triggering their maturation and survival, but also indirectly interfere with B cell immunity by dysregulating innate immune responses and T cell activation and balance. | BAFF (Regulating naïve B cell) |

**Abbreviations:**

**USM B cells:** un-class-switched memory B cells

**Bregs:** regulatory B cells

**PD:** Parkinson's disease

**HDs:** Healthy donors

**BAFF：**B cell activating factor of the tumor necrosis factor family

**IL:** Interleukin
